# A case report of hearing loss post use of hydroxychloroquine in a HIV-infected patient

**DOI:** 10.1186/2008-2231-22-20

**Published:** 2014-01-22

**Authors:** Hossein Khalili, Farzaneh Dastan, Seyed Ali Dehghan Manshadi

**Affiliations:** 1 Department of Clinical Pharmacy, Faculty of Pharmacy, Tehran University of Medical Sciences, Tehran 1417614411, Iran; 2 Department of Infectious Diseases, Faculty of Medicine, Tehran University of Medical Sciences, Tehran, Iran

**Keywords:** Acquired immunodeficiency syndrome, Case report, Hearing loss, Human immunodeficiency virus, Hydroxychloroquine

## Abstract

**Objective:**

A case with reversible symmetrical sensorineural hearing loss following hydroxychloroquine therapy is described.

**Case summary:**

A 57-year-old, human immunodeficiency virus (HIV) positive man was referred to the HIV clinic of Imam Khomeini Hospital, Tehran with chief complaint of bilateral slowly progressive hearing loss starting from two months ago. The man had history of rheumatoid arthritis diagnosed from 3 months ago and was administered hydroxychloroquine 200 mg and prednisolone 5 mg twice daily. Audiometry test showed moderate to severe neuronal hearing loss and reduced speech recognition in both ears of the patient. With suspicion of hydroxychloroquine-induced hearing loss, this drug was discontinued. After 2 months of hydroxychloroquine discontinuation, his audiometry findings were improved.

**Discussion:**

A few cases of hydroxychloroquine-induced hearing loss have been reported. All of the cases were non-HIV positive individuals. Irreversible hearing loss was developed following long-term therapy with hydroxychloroquine. The present case was a HIV-positive man who developed hearing loss following short course (one month) hydroxychloroquine therapy and his problem was resolved following discontinuation of hydroxychloroquine and continuation of prednisolone.

**Conclusions:**

Hydroxychloroquine-induced hearing loss may reversibly occur following short term therapy in HIV patients.

## Introduction

Hydroxychloroquine (HQ), a quinoline compound, rarely causes ototoxicity. Its ototoxicity is associated with varying degree of destruction of the cochlear sensory hair cells, a decrease in neuronal population, alteration in supporting structures and atrophy and vacuolization of the stria vascularis as a possible consequence of ischemia [1,2]. Clinically significant HQ-related adverse reactions including retinopathy and other visual disorders are usually detected during long-term therapy [3]. Deafness following prolonged therapy with HQ has been also reported [4]. In the previous report, ototoxicity of HQ was irreversible and manifested by auditory dysfunction without vestibular changes [5]. In the present case, HQ-induced bilateral reversible hearing loss is described in a HIV positive man suffering from rheumatoid arthritis (RA).

## Case report

A 57-year-old HIV positive man was referred to the HIV clinic of Imam Khomeini Hospital, Tehran, Iran, with chief complaint of bilateral slowly progressive hearing loss starting from two months ago. He had no previous cochleo-vestibular symptoms and the hearing loss was described without tinnitus, vertigo or balance changes. He had no history of head trauma prior to the beginning of the hearing loss. He was a known case of HIV infection following blood transfusion from 2 years ago. In his medical history, RA was diagnosed from 3 months ago when he was receiving HQ 200 mg and prednisolone 5 mg twice daily. He did not consume any antiretroviral supplements or herbal products.

His laboratory findings showed CD4 count of 107/L and CD4/CD8 ratio of 0.13. All the other routine laboratory parameters were within the normal range. Based on the patient clinical status and his CD4 count, antiretroviral regimen including lamivudine, zidovudine and efavirenz was started. Also sulfamethoxazole/trimethoprim and isoniazid was considered for prophylaxis of pneumocystis and tuberculosis respectively in this patient.

**Figure 1:**
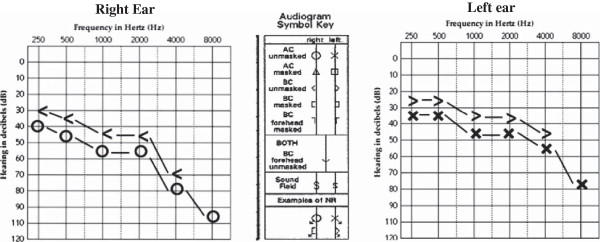
The audiogram of the patient after 1 month of treatment with hydroxychloroquine.

Otolaryngological consultation reported normal otoscopic and neurologic examinations for the patient. Pure-tone (air and bone conduction) and speech audiometry showed moderate to severe neuronal hearing loss and reduced speech recognition in his both ears (45 and 40 dB in the right and left ears respectively) (Figure 1). Absence of middle ear pathologic conditions was confirmed by pneumatic otoscopy and tympanometry. Furthermore, acoustic reflexes were latent in the patient.

**Figure 2:**
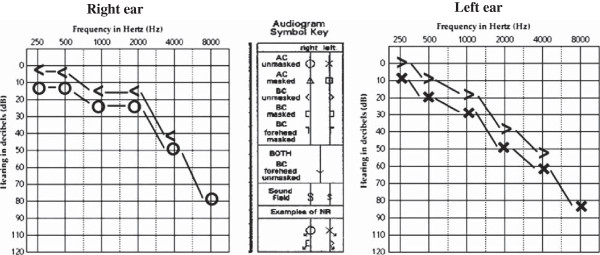
The audiogram of the patient, 2 months after discontinuation of hydroxychloroquine.

With suspicious of HQ-induced hearing loss, the drug was discontinued and prednisolone was continued to control his RA symptoms. Two months later, his audiometric findings improved. Pure-tone and speech audiometry revealed mild to moderate hearing loss and slight to mild disability in speech recognition in the right and left ears, respectively (Figure 2). His acoustic reflexes were still latent.

## Discussion

Idiopathic sudden sensorineural hearing loss usually occurs 5–20 per 100,000 populations mostly due to viral infections, vascular occlusion with microcirculatory disturbances, immunologic diseases, or intralabyrinthine membrane breaks [6]. Some drugs may cause vestibulocochlear toxicity. Chloroquine another quinoline compound aggregates in melanocytes and results in variable injuries to the cochlear sensory hair cells, decrease in neuronal population, loss of supporting hair cells, and atrophy of stria vascularis. These changes might be caused by an ischemic process [1,7]. There are some reports about chloroquine-induced ototoxicity [7-9]. Severe chloroquine-induced cochleovestibular toxicity was reported in a pregnant woman [8]. Absence of inner and outer hair cells of the cochlea was also detected in a child whose mother took chloroquine during her pregnancy [9]. Scherbel *et al.* reported tinnitus, a sense of imbalance and nerve deafness after prolonged chloroquine administration [7].

Only a few cases of HQ-induced hearing loss have been reported. First case was a 44-year-old woman and the second case was a 44-year-old man with lupus erythematous. Both of these patients developed irreversible hearing loss following several years of HQ treatment [5]. The third patient was a 34-year-old woman with diagnosis of RA, who developed reversible, bilateral hearing loss following five months of HQ therapy [10]. Another report was unilateral sensorineural hearing loss in a 7-year-old girl with idiopathic pulmonary hamosiderosis. Her problem was diagnosed after 2 years of HQ administration [11].

All of the previous cases were non-HIV positive individuals. In these patients HQ-induced ototoxicity developed following long term HQ administration. In most of them, hearing loss was irreversible. Our case was a HIV-positive man who developed hearing loss following short course (one month) HQ therapy and his problem was resolved following HQ discontinuation.

HQ is structurally related to chloroquine and shows similar ototoxicity pattern. Chloroquine-induced hearing loss is reversible if prompt chloroquine cessation and steroid administration is done [12]. Our patient was receiving prednisolone concomitant with HQ and it is justifiable to suppose that the reversibility of his hearing loss was due to concomitant administration of this anti-inflammatory agent.

Our case is the first report of reversible symmetrical hearing loss following HQ therapy in a HIV-infected patient. The patient received daily HQ with dose of 200 mg twice daily for 3 months. No other causes of hearing loss were detected in this patient. Hearing loss in HIV infected persons may result from opportunistic infections such as cryptococcal meningitis or neurosyphilis [13] and ototoxic medications [14]. Although hearing loss was reported following antiretroviral therapy [15], in our patient these drugs were started two months after the beginning of his hearing loss. There is no any difference in audiometric findings in patients with RA compared with non-RA subjects [16].

According to the Naranjo probability scale [17], hearing loss in the present patient was probably related to HQ administration. According to definition of adverse reaction severity [18], that reaction was categorized as grade 3.

Corticosteroids are only confirmed effective treatment of sudden sensorineural hearing loss. It is important to notice that prednisone should be started as soon as possible after the onset of hearing loss [19].

In conclusion HQ-induced hearing loss may appear following short-term administration especially in patients with underlying viral infections and may be reversible by corticosteroid therapy.

### Consent

Written informed consent was obtained from the patient for the publication of this report and any accompanying images.

## Competing interests

The authors declare that they have no competing interests.

## Authors’ contribution

HK: Followed the case and edited the manuscript. FD: The case was detected and followed by FD in HIV clinic of the hospital. She also drafted the manuscript. AD: Did clinical assessment of the patient. All authors read and approved the final manuscript.
